# Improving coriander yield and quality with a beneficial bacterium

**DOI:** 10.1186/s43897-024-00087-2

**Published:** 2024-02-29

**Authors:** Xiaoxuan Wu, Yu Yang, Miao Wang, Chuyang Shao, Juan I. V. Morillas, Fengtong Yuan, Jie liu, Huiming Zhang

**Affiliations:** 1 Shanghai Center for Plant Stress Biology, Center for Excellence in Molecular Plant Sciences, Chinese Academy of Sciences, Shanghai, 201602 China; 2 University of Chinese Academy of Sciences, Beijing, 100049 China; 3 Instituto de Tecnologia Química e Biológica (ITQB), Oeiras, Lisbon, Portugal; 4 Nanchang Institute of Industrial Innovation, Chinese Academy of Sciences, Nanchang, 330224 China; 5 Jiangxi Center for Innovation and Incubation of Industrial Technologies, Chinese Academy of Sciences, Nanchang, 330200 China; 6 Institute of Geographic Sciences and Natural Resources Research, Chinese Academy of Sciences, Beijing, 100101 China

An important trend in global agriculture is to utilize plant probiotics, which are microbes that can promote plant growth and/or increase plant resistance to stress conditions, to reduce the use of chemical fertilizers and pesticides while maintaining or increasing yield [1, 2]. The global market size of plant probiotics for crops was valued at USD 5. 27 billion in 2021 and is projected to reach 15.71 billion by 2029 (http://www.researchandmarkets.com/reports/5165433/agricultural-microbial-market-growth-trends). Probiotic-induced plant growth promotion commonly involves improvements in nutrient acquisition and the modulation of phytohormone homeostasis [1], whereas the potential contributions of other cellular processes, such as the biogenesis of ribosomes, which translates mRNAs into proteins and accordingly has an essential role in the control of cell growth, have received little attention. In addition, unlike the apparent effects on plant growth, the effects of probiotics on plant secondary metabolites at the omics level, which are important for the quality of plant-based foods, are generally unclear.

Coriander (*Coriandrum sativum* L.), commonly known as cilantro or Chinese parsley, is a worldwide culinary and medicinal plant with both nutritional and medicinal properties [3, 4]. The leaves of juvenile coriander plants are popular in cuisine for their distinctive aroma. Little is known about how plant probiotics may promote growth or affect the food chemistry profile of coriander plants. The soil bacterium *Aeromonas* sp. H1 is a recently isolated probiotic strain effective for many plant species [5]. In this study, we investigated how the yield and quality of coriander may be affected by *Aeromonas* sp. H1 using transcriptomic and metabolomic approaches.

*Aeromonas* sp. H1 significantly increased the biomass of coriander, as indicated by a 34.1% increase in the aerial fresh weight at 14 days after treatment (DAT) (Fig. 1A and B; Fig. S1A). The H1-treated coriander also presented increased levels of soluble proteins and soluble sugars but not chlorophylls or carotenoids (Fig. 1C; Fig. S1B, C). To gain mechanistic insights into H1-induced growth promotion, we profiled the coriander transcriptome via mRNA sequencing of the aerial parts of plants at 8-DAT when growth promotion was about to become apparent, with the rationale that transcriptional regulation precedes the resultant morphological changes. A total of 1123 differentially expressed genes (DEGs, treated vs. control, fold change ≥ 1.5, FDR ≤ 0.05) were identified (Supplementary Table S1; Fig. S1D), including 405 downregulated genes and 718 upregulated genes. Gene Ontology (GO) analysis of the downregulated DEGs highlighted the biological process of autophagy (Fig. 1D; Supplementary Table S2). This observation suggested that the H1-treated plants were healthier than the control plants, given that autophagy is responsible for recycling dysfunctional cellular components [6]. GO analysis revealed that the DEGs were characterized by the upregulation of ribosome biogenesis (Fig. 1D; Supplementary Table S2), which included the large subunit and small subunit ribosomal proteins and regulators of ribosome biogenesis (Fig. 1E; Supplementary Table S1); together, these DEGs accounted for 22.9% of all the DEGs and 34.8% of all the ribosome biogenesis genes in coriander; these ratios were remarkable since the ratio of all DEGs to all genes in the coriander genome was only 4.6%. The transcriptional elevation of ribosome biogenesis DEGs was observed at 8-DAT but not 4-DAT or 14-DAT (Fig. 1F), indicating that transcriptional regulation is a hallmark event at the onset of H1-induced growth promotion. In addition to the ribosome biogenesis DEGs, the DEGs involved in tRNA synthesis, translation initiation, and translation elongation were also predominantly upregulated (Fig. S1E, Supplementary Table S1). Thus, the H1-induced growth promotion in coriander was underpinned by the bacterial enhancement of ribosome biogenesis and protein production.

**Fig. 1:**
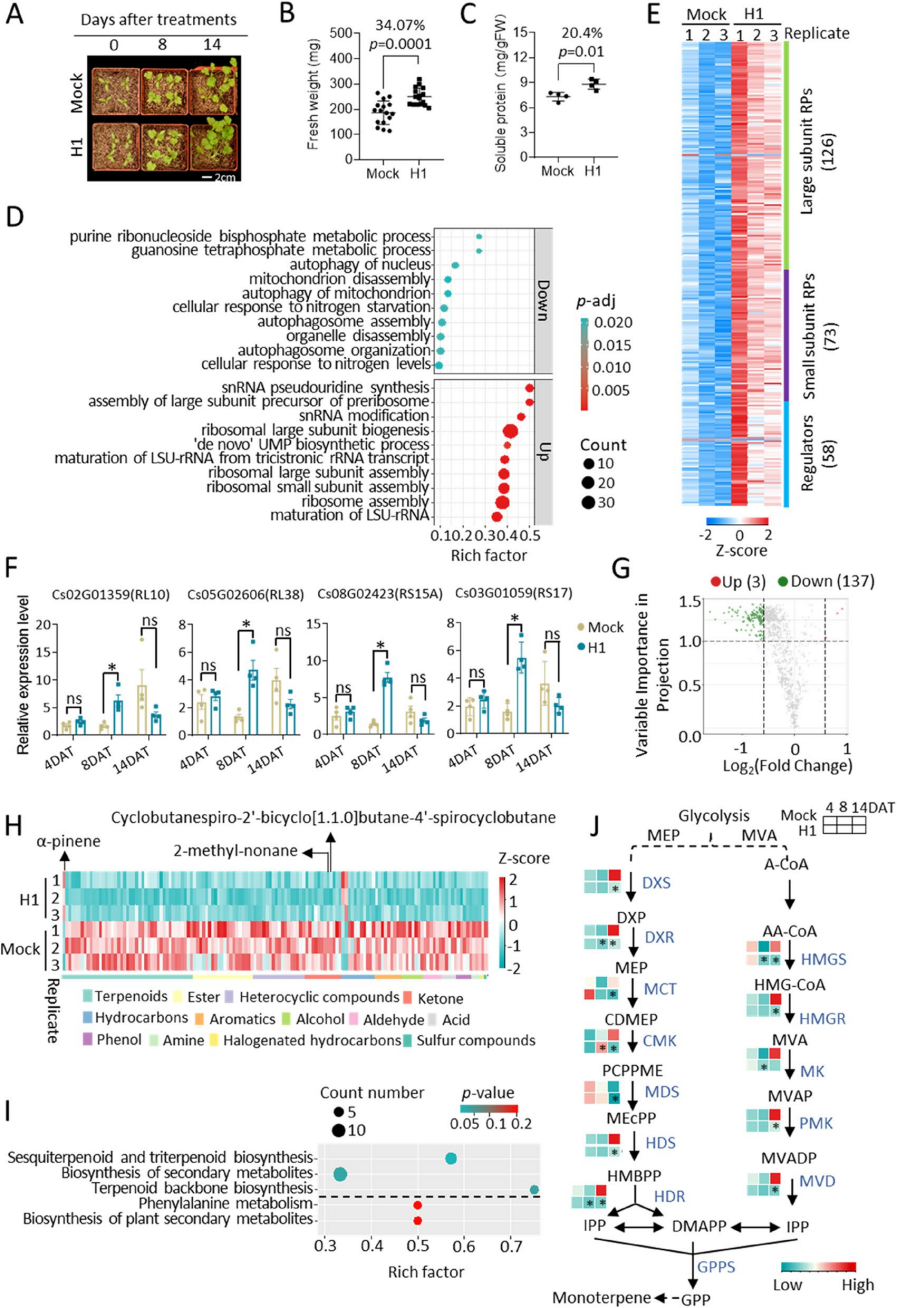
Transcriptomic and metabolomic insights into the effects of *Aeromonas* sp. H1 on the yield and quality of coriander.** A** and **B** H1 increased the biomass of coriander. Data are presented as mean ± SD with Student’s t-test *p-*value, *n* = 16 biological replicates. **C** H1 increased the content of soluble proteins in coriander at 14 DAT. Mean ± SD with Student’s t test *p* value; *n* = 4 biological replicates. **D** The top 10 GO biological processes enriched in the down- and upregulated DEGs. **E** A heatmap showing the transcriptional activation of ribosome biogenesis in H1-treated coriander. RNAseq results, 3 biological replicates. **F** Time course gene expression patterns of representative ribosome biogenesis DEGs. The RT‒qPCR data are shown relative to those of the 8-DAT mock sample, mean ± SD, *n* = 4, asterisks (*) indicate *p* < 0.05, Student’s t-test. **G** A volcano plot showing the distribution patterns of the volatile metabolome of coriander leaves at 14-DAT. Differentially accumulated metabolites (DAMs) between samples were defined by a fold change ≥ 1.5 and VIP ≥ 1.0; *n* = 3 biological replicates. **H** A heatmap of H1-induced DAMs in coriander. Arrows indicate the names of the three upregulated metabolites. **I** KEGG enrichment analysis of H1-induced DAMs. Groups above the dashed lines are those with a *p* value < 0.05. **J** The expression of genes in the terpene backbone synthesis pathways at 4-, 8-, and 14-DAT. RT‒qPCR values are shown relative to those of *HMGS* in the H1-treated 14-DAT sample. The full names of the genes and metabolites are listed in Supplementary Table S4. Asterisks indicate significant differences between the mock and H1 groups at each time point. *n* = 4, *p* < 0.05, Student’s t test

Coriander leaves have a distinctive aroma and are enriched in a variety of secondary metabolites [3, 4, 7]. To determine whether H1 altered the quality of coriander, we profiled the volatile metabolome of coriander leaves at 14 DAT. The volatile organic compounds (VOCs) were collected from the samples by using solid-phase microextraction (SPME), followed by gas chromatography coupled with mass spectrometry (GC‒MS) analysis. A total of 855 VOC components were identified in the coriander metabolome; 3 and 137 of these compounds exhibited increased and decreased levels, respectively (fold change ≥ 1.5, VIP ≥ 1), as a result of H1 inoculation (Fig. 1G; Fig. S1F; Supplementary Table S3). Terpenoids accounted for the largest group (30.7%) of the decreased metabolites (Fig. 1H); moreover, Kyoto Encyclopedia of Genes and Genomes (KEGG) pathway analysis revealed that the decreased metabolites were particularly enriched in terpenoid biosynthesis (Fig. 1I), including a group of sesquiterpenoids and triterpenoids (Fig. S1G). Terpenoid backbones are synthesized from two universal precursors, isopentenyl pyrophosphate (IPP) and its isomer dimethylallyl pyrophosphate (DMAPP) [8]. Consistent with the metabolic repression of terpenoid backbone biosynthesis, the H1-treated coriander at 14-DAT presented repressed gene expression of DXR (1-deoxy-D-xylulose 5-phosphate reductoisomerase) and HMGR (hydroxymethylglutaryl-CoA reductase) (Fig. 1J), which are rate-limiting enzymes in the biosynthetic pathways for IPP and DMAPP, respectively [8]. H1-treated coriander also showed transcriptional repression of isoprene synthase (Fig. S1H, I), which produces isoprene, although isoprene was not detected in the metabolome, likely because isoprene biosynthesized in situ by isoprene synthase is immediately released and not stored in the leaf [9]. Interestingly, despite the overall suppression of terpenoid biosynthesis (Fig. 1J), H1-treated coriander plants exhibited significantly increased levels of α-pinene (Fig. 1H), a monoterpene of intense medicinal interest due to its antimicrobial, antioxidant, anti-inflammatory, and neuroprotective activities [10]. In coriander, unsaturated aldehydes, mainly decanal and dodecanal, are described by many people as fruity, green and pungent, while (E)-2-alkenals, mainly (E)-2-decenal and (E)-2-dodecenal, taste soapy to some other people due to genetic variants in olfactory receptors [11]. The H1-treated coriander plants produced similar levels of both fruity and soapy metabolites as did the control plants (Fig. S1J).

In conclusion, the application of *Aeromonas* sp. H1 not only increased the yield but also the contents of soluble proteins and α-pinene in coriander without altering its known flavors. These findings demonstrate the promising ability of plant probiotics to improve the nutritional and medicinal qualities of plants in agriculture and horticulture. This study also highlights that probiotic-induced plant growth promotion can be supported by the enhancement of ribosome biogenesis, as indicated by the strong enrichment of ribosome biogenesis-related DEGs in the coriander transcriptome, such as *Cs05G02606* and *Cs08G02423*, which encode the *60 S ribosomal protein L38* (*RL38*) and the *40 S ribosomal protein S15a* (*RS15A*), respectively. While the functional genomics of coriander is currently limited, future research involving in-depth functional verification of the key regulatory genes will help improve our understanding of metabolic regulation in coriander.

### Supplementary Information

**Additional file 1:** [1] Materials and Methods [2] Figure S1.

**Additional file 2:** Supplementary Table S1-S5.

## Data Availability

The raw RNA-seq data are available in the NCBI GEO (http://www.ncbi.nlm.nih.gov/geo/) under accession number GSE247080.
