# Supplementary material for: Improving coriander yield and quality with a beneficial bacterium
Source: Mol Hortic. 2024 Feb 29;4:8. doi: 10.1186/s43897-024-00087-2 (PMC10903023; doi:10.1186/s43897-024-00087-2)
Supplement: Supplementary file 1 — Additional file 1: [1] Materials and Methods [2] Figure S1. [file 43897_2024_87_MOESM1_ESM.docx]

**Improving coriander yield and quality with a beneficial bacterium**

Xiaoxuan Wu^1,2^, Yu Yang^1^, Miao Wang^1,2^, Chuyang Shao^1^, Juan I.V. Morillas^3^ Fengtong Yuan^4,5^, Jie liu^6^, Huiming Zhang^1,4,5^ *

**Affiliations**

^1^ Shanghai Center for Plant Stress Biology, Center for Excellence in Molecular Plant Sciences, Chinese Academy of Sciences, Shanghai 201602, China

^2^ University of Chinese Academy of Sciences, Beijing 100049, China

^3^ Instituto de Tecnologia Química e Biológica (ITQB), Oeiras, Lisbon, Portugal

^4^ Nanchang Institute of Industrial Innovation, Chinese Academy of Sciences, Nanchang 330224, China

^5^ Jiangxi Center for Innovation and Incubation of Industrial Technologies, Chinese Academy of Sciences, Nanchang 330200, China

^6^ Institute of Geographic Sciences and Natural Resources Research, Chinese Academy of Sciences, Beijing 100101, China

* Correspondence: H. Zhang ([hmzhang@psc.ac.cn](mailto:hmzhang@psc.ac.cn))

**Email addresses of all the authors**

Xiaoxuan Wu: xxwu@psc.ac.cn

Yu Yang: yuyang@psc.ac.cn

Miao Wang: wangmiao@cemps.ac.cn

Chuyang Shao: [cyshao@psc.ac.cn](mailto:cyshao@psc.ac.cn)

Juan I.V. Morillas: nacho.vilchez@itqb.unl.pt

Fengtong Yuan: ftyuan@sibs.ac.cn

Jie Liu: liujie@cashq.ac.cn

Huiming Zhang: [hmzhang@psc.ac.cn](mailto:hmzhang@psc.ac.cn); Tel: 86-21-57078248; Fax: 86-21-54924015

**Supplementary Materials**

[1] Materials and Methods

[2] Figure S1

**[1] Materials and Methods**

**Plant Materials and Growth Conditions**

The seeds of coriander (*Coriandrum sativum* L.) were sown in 0.4 liter pots filled with mixed soil (pindstrup substrate: vermiculite; 1:1 in volume) that had been tyndallized (75°C for 3 h plus a cooling interval of 10 h plus 75°C for another 3 h) and subsequently grown in a growth room at 22°C with a 16 h light/8 h night cycle. Nine-day-old coriander plants of similar size were transferred to new pots and grown under the same conditions for another 7 days before bacterial inoculation.

**Bacterial growth and inoculum preparation**

The bacterial strain *Aeromanas sp*. H1 was isolated in a previous study (He et al. 2022). For bacterial inoculation, the bacteria were streaked on Luria–Bertani (LB) plates, and a single colony from the LB plate was cultured overnight in liquid LB medium (28°C; 250 rpm) to reach the exponential growth phase, as estimated by the optical density measured at a wavelength of 600 nm (OD600) approximately equal to 0.8–1.1. The bacterial culture was centrifuged at 4000 rpm for 20 min in an Avanti J-E High-Speed Centrifuge (Beckman Coulter) and resuspended in 0.45% NaCl for soil inoculation. The mock control was conducted by using 0.45% NaCl solution without bacteria.

**Bacterial colonization test**

The bacterial colonization test was performed as previously described with minor modifications (Vílchez et al. 2020). In detail, plant roots were separated from the aerial parts and collected 1 day after bacterial inoculation. Roots from 4 plants were harvested as 1 biological replicate, and each sample included 5 biological replicates. The collected roots were surface-sterilized in 75% ethanol for 50 seconds and then washed with sterilized water 6 times. The washed roots were collected in preweighed tubes, and the fresh weights of the roots were recorded. Sterilized 0.45% NaCl was added to the tubes at three times the volume of the fresh weight (V/W) of the roots. The roots were then homogenized by TissueLyser (Retsch) at a frequency of 25 Hz. Serial dilutions were prepared using sterilized 0.45% NaCl and spread on LB plates. After incubating at 28°C overnight, the colonies were counted, and the number of colony-forming units (CFU) per mg of the root fresh weight was calculated.

**RNA-seq and data analyses**

The aerial parts of the coriander plants were harvested at 8 days after H1 treatment (DAT), and total RNA was extracted using the Eastep Super Total RNA Extraction Kit (Promega). The aerial parts from 4 plants were harvested as 1 biological replicate, and each sample contained 3 biological replicates. Libary preparation and sequencing were performed by MetWare Biotechnology, Inc. (Wuhan, China). In brief, RNA quality was assessed using the RNA Nano 6000 Assay Kit of the Bioanalyzer 2100 system (Agilent) and the NanoPhotometer spectrophotometer (IMPLEN). The RNA concentration was measured using a Qubit RNA Assay Kit in a Qubit 2.0 Fluorometer (Life Technologies). A total of 1 μg of RNA per sample was used as input material for the library preparations using the NEBNext UltraTM RNA Library Prep Kit for Illumina (NEB) following the manufacturer’s recommendations. Sequencing was performed on an Illumina PE150 platform.

For RNA-seq data analysis, the raw data were preprocessed with fastp v 0.19.3, and the clean reads were subsequently aligned to the reference genome using HISAT v2.1.0. The reference genome of the coriander was downloaded from CGDB (<http://cgdb.bio2db.com/>) (Song et al. 2020). FeatureCounts v1.6.2 was used to calculate the gene alignment and FPKM values. DESeq2 v1.22.1 was used to analyze the differential expression between the two groups, and the P value was corrected using the Benjamini & Hochberg method. A corrected P value ≤ 0.05 and a fold change ≥ 1.5 were used as the thresholds for significantly different expression. The GO enrichment analyses were performed using the online tool MetWare Cloud (<https://cloud.metware.cn>). The heatmaps were generated with TBtools software (Chen et al. 2023).

**Volatile metabolome measurements and data analyses**

For plant volatile metabolome measurements, the aerial parts of coriander plants were harvested at 14 days after H1 (DAT) treatment and frozen in liquid nitrogen immediately. Twelve plants were harvested as 1 biological replicate, and each sample included 3 biological replicates. The profiling of the volatile metabolome was performed by MetWare Biotechnology, Inc. (Wuhan, China). In brief, the samples were ground into powder in liquid nitrogen. Five hundred milligrams of the powder was transferred immediately to a 20 mL head-space vial (Agilent) containing NaCl saturated solution to inhibit any enzymatic reaction. The vials were sealed using crimp-top caps with TFE-silicone headspace septa (Agilent). For SPME analysis, each vial was heated at 60°C for 5 min, after which a 120 µm DVB/CWR/PDMS fiber (Agilent) was exposed to the headspace of the sample for 15 min at 60°C.

After sampling, desorption of the VOCs from the fiber coating was carried out in the injection port of the GC apparatus (Model 8890; Agilent) at 250°C for 5 min in splitless mode. The identification and quantification of VOCs were carried out using an Agilent Model 8890 GC and a 7000D mass spectrometer (Agilent) equipped with a 30 m × 0.25 mm × 0.25 μm DB-5MS (5% phenyl-polymethylsiloxane) capillary column. Helium was used as the carrier gas at a linear velocity of 1.2 mL/min. The injector temperature was kept at 250°C, and the detector temperature was held at 280°C. The oven temperature was programmed from 40°C (3.5 min), increased at 10°C/min to 100°C, increased at 7°C/min to 180°C, and increased at 25°C/min to 280°C for 5 min. Mass spectra were recorded in electron impact (EI) ionization mode at 70 eV. The quadrupole mass detector, ion source and transfer line temperatures were set at 150°C, 230°C and 280°C, respectively. The selected ion monitoring (SIM) mode was used for the identification and quantification of analytes. Three Quality control (QC) samples were tested at the beginning, in the middle and at the end of the run for the samples to be tested. A QC sample was a mixture of equal volume aliquots of all samples to be tested and processed in the same manner for GC-MS analysis.

The MS raw data were processed with MassHunter software for qualitative and quantitative analyses based on the MetWare self-established database. Unsupervised principal component analysis (PCA) was performed with the statistical function prcomp within R (www.r-project.org). The data were subjected to unit variance scaling before unsupervised PCA. Differentially accumulated metabolites (DAMs) were determined by variable importance in projection (VIP ≥ 1) and fold change (fold change ≥ 1.5). VIP values were extracted from OPLS-DA (OSC partial least squares-discriminant analysis) results generated by the R package MetaboAnalystR. The data were log-transformed (log_2_) and mean centered before OPLS-DA. The identified metabolites were annotated using the KEGG Compound database (<http://www.kegg.jp/kegg/compound/>), and the annotated metabolites were subsequently mapped to the KEGG Pathway database (<http://www.kegg.jp/kegg/pathway.html>). KEGG enrichment analysis was performed by using the MetWare Cloud. Heatmaps were generated with TBtools software.

**Quantitative real-time PCR**

Total RNA was extracted from the aerial parts of plants at the indicated time points using an Eastep Super Total RNA Extraction Kit (Promega). One microgram of total RNA was subjected to reverse transcription using HiScript III RT SuperMix for qPCR (+gDNA wiper) (Vazyme) in a 20 μL volume according to the manufacturer’s instructions. Q-PCR was performed using the ChamQ Universal SYBR qPCR Master Mix (Vazyme) in a 10 μL volume using the QuantStudio (TM) 6 Flex System (Applied Biosystems). The PCR was programed as: initial denaturation of 95°C for 3 min; an amplification phase of 40 cycles with a denaturation phase of 95°C (10 s); and an annealing phase at 60°C (45 s). The gene *Cs06G02549* (*GAPDH*) was used as the internal control. All the primers used are listed in Supplementary Table S5.

**Measurements of multiple traits for plant growth promotion**

Plant growth promotion was quantified 14 days after H1 inoculation. The aerial parts of the coriander plants were harvested and weighed for fresh weight quantification. For chlorophyll and carotenoid content measurements, leaves from 4 plants were harvested as 1 biological replicate, and each sample contained 6 biological replicates. Approximately 100 mg of leaf tissue was immersed in 1 mL of ethanol overnight in darkness until the leaves became fully white. Then, the optical density (OD) at 664 nm, 648 nm, and 470 nm of the extract was read using a Thermo Varioskan Flash Microplate Reader. The chlorophyll content was calculated by the following equation: chlorophyll a concentration (Ca) = 13.95 × OD664 – 6.88 × OD648; chlorophyll b concentration (Cb) = 24.96 × OD648 - 7.32 × OD664; total chlorophyll concentration (CT) = Ca+Cb; and carotenoid concentration = (1000 × OD470 – 2.05 × Ca-114.8 × Cb)/245; and content (mg/g) = concentration (mg/L) × 1 mL/fresh weight (mg).

For soluble sugar content measurements, aerial parts from 4 plants were harvested as 1 biological replicate, and each sample contained 5 biological replicates. The aerial parts were cut into pieces, and approximately 100 mg of fresh material was further ground with 1 mL of distilled water. Then, the homogenate was boiled for 10 min. After cooling, the homogenate was centrifuged at 8000 × g for 10 min at room temperature. The supernatant was subsequently transferred to a 50 mL tube, which was subsequently filled with distilled water to a volume of 20 mL. The soluble sugar concentration was quantified based on a standard curve using a Plant Soluble Sugar Content Assay Kit (Solarbio) following the manufacturer’s instructions. A standard curve was generated with serially diluted glucose standards (0.006, 0.013, 0.025, 0.05, 0.1, and 0.2 mg/mL), and the corresponding OD was determined at 620 nm. The OD at 620 nm was read using a Thermo Varioskan Flash Microplate Reader. Content (mg/g FW) = 20 mL × concentration (mg/mL)/FW (g).

For soluble protein content measurements, aerial parts from 12 plants were harvested as 1 biological replicate, and each sample contained 4 biological replicates. The samples were ground into powder in liquid nitrogen, and approximately 50 mg of powder was homogenized in 100 μL of protein extraction buffer [20 mM Tris-HCl (pH=7.5), 5 mM EDTA, 150 mM NaCl, 2 mM Dithiothreitol, and protease inhibitor cocktail (Roche Applied Science)]. After centrifugation at 13,523 × g for 30 min at 4°C, the supernatant was collected, and the concentration was determined based on the standard curve by using Pierce BCA Protein Assay Kits (Thermo Scientific) according to the manufacturer’s instructions. A standard curve was generated with serially diluted albumin (BSA) standards (0, 25, 125, 250, 500, 750, 1000, 1500, 2000 μg/mL), and the corresponding OD was measured at 562 nm. The OD at 562 nm was read using a Thermo Varioskan Flash Microplate Reader. The soluble protein content (mg/g FW) was calculated as 0.1 mL × concentration (μg/mL)/FW (mg).

**Statistical analysis**

Statistical analyses for pairwise group comparisons were performed with Student’s t test using Excel. Differences were considered significant at p ≤ 0.05. The scatter plots and bar graphs were generated using GraphPad Prism 8.0.1.

**Method reference**

Chen, C., Y. Wu, et al. (2023). "TBtools-II: A "one for all, all for one" bioinformatics platform for biological big-data mining." Mol Plant.

He, D., S. K. Singh, et al. (2022). "Flavonoid-attracted Aeromonas sp. from the Arabidopsis root microbiome enhances plant dehydration resistance." ISME J **16**(11): 2622-2632.

Song, X., F. Nie, et al. (2020). "Coriander Genomics Database: a genomic, transcriptomic, and metabolic database for coriander." Hortic Res **7**: 55.

Vílchez, J. I., Y. Yang, et al. (2020). "DNA demethylases are required for myo-inositol-mediated mutualism between plants and beneficial rhizobacteria." Nature Plants **6**(8): 983.

**[2] Figure S1. *Aeromonas* sp. H1 increased the yield and quality of coriander without affecting its flavor.**


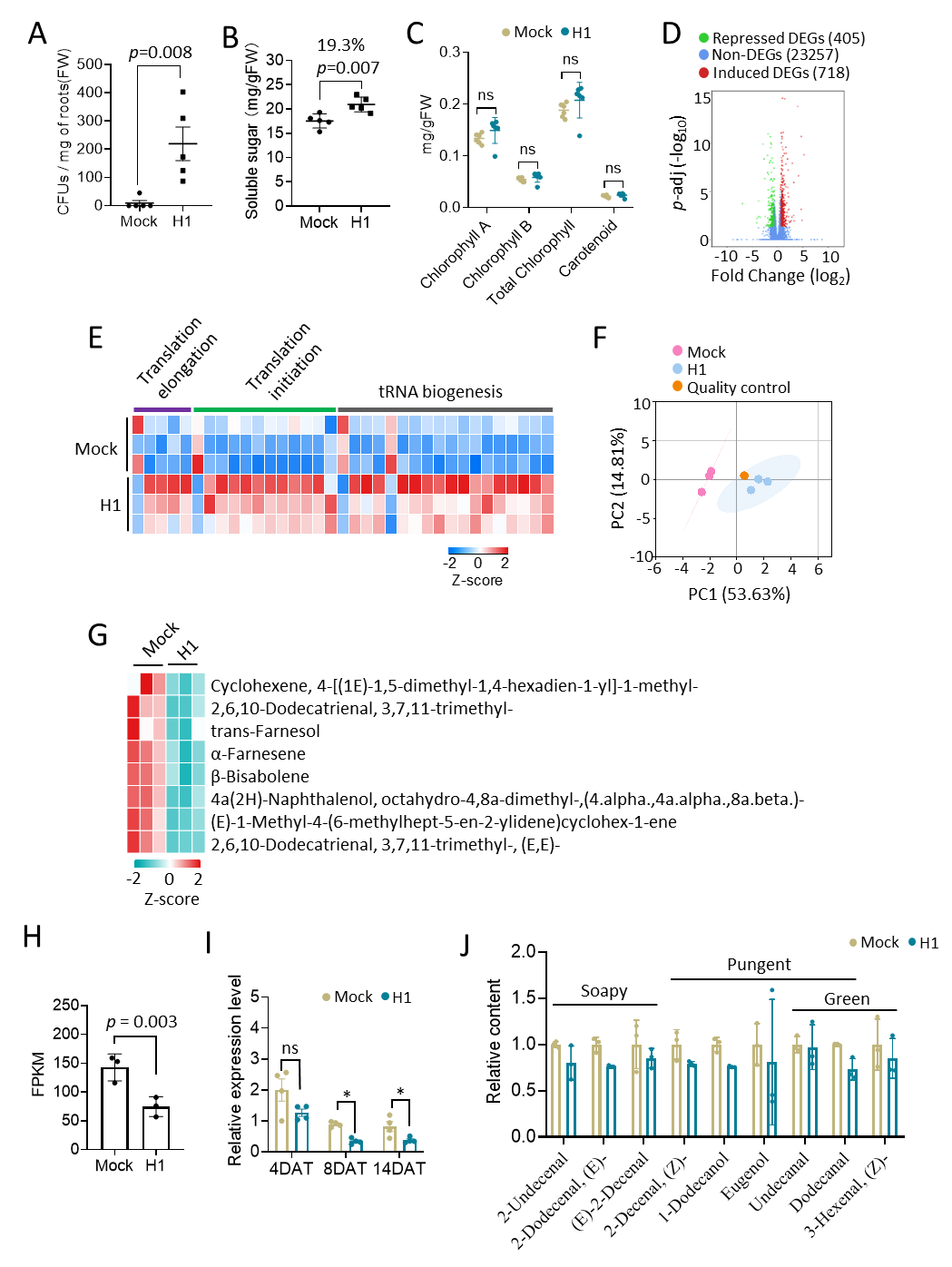


**Figure S1. *Aeromonas* sp. H1 increased the yield and quality of coriander without affecting its unique flavor. [A]** Coriander root colonization of H1 at 14-DAT. Mean ± SE with Student's t test *p* value; n = 5 biological replicates. **[B]** and **[C]** H1 increased the content of soluble sugars (n=5) but not that of chlorophylls or carotenoids (n=6) in coriander at 14-DAT. Mean ± SD with Student's t test *p* value; ns indicates nonsignificance; n = biological replicates. **[D]** A volcano plot showing the distribution patterns of DEGs and non-DEGs. mRNA sequencing results, n=3 biological replicates. **[E]** Heatmap of the DEGs encoding tRNA synthesis, translation initiation, and translation elongation. **[F]** Principal component analysis (PCA) of the volatile metabolome separated the H1-treated and mock samples into different clusters. **[G]** A heatmap of the differentially accumulated metabolites (DAMs) belonging to the KEGG pathway of sesquiterpenoid and triterpenoid biosynthesis. **[H]** H1 repressed the gene expression of isoprene synthase in coriander at 8-DAT, as detected by RNA-seq **[I]** H1 repressed the gene expression of isoprene synthase in coriander at 8-DAT and 14-DAT, as detected by RT‒qPCR. **[J]** H1-treated coriander showed similar levels of the characteristic odorants as the control plants. Data extracted from the volatile metabolome.
